# Congenital Intrathoracic Ectopic Kidney in Association With Bochdalek Hernia

**DOI:** 10.7759/cureus.22674

**Published:** 2022-02-28

**Authors:** Mahmoud Al Eraky, Lamma Hassan, Ahmed Abou Zaid, Hisham Arabi

**Affiliations:** 1 Pediatrics Department, King Abdullah Specialist Children Hospital (KASCH), Riyadh, SAU; 2 Medical Education, College of Medicine, Alfaisal University, Riyadh, SAU

**Keywords:** intrathoracic, bochdalek hernia, congenital diaphragmatic hernia, pediatric case report, ectopic kidney

## Abstract

Thoracic ectopic kidney is the rarest type of ectopic kidney with a reported finding of one in every 15,000 autopsies. The diagnosis of this anomaly is often incidental. Children may be symptomatic with recurrent respiratory symptoms. We present a case of an infant with a thoracic ectopic kidney associated with a congenital diaphragmatic hernia that was initially misdiagnosed as unresolved pneumonia due to persistent well-demarcated opacity in the left lower lobe. Our aim is to increase awareness about this rare entity.

## Introduction

Ectopic kidney is a rare congenital anomaly in which one or both kidneys are located outside of the iliac fossa. The incidence is one in 1000 live births. Ectopic kidney can be located in the pelvis, ileum, abdomen, or thorax. The most common location of the ectopic kidney is the pelvis, with the thorax being the rarest. Ectopic thoracic kidney constitutes <5% of all renal ectopias [1]. It is more common in males than females (2:1), and has a slight left side predominance [2]. This is partially explained by the fact that the liver acts as a physical barrier to the right hemidiaphragm [3].

In most cases, the ectopic thoracic kidney is asymptomatic and is diagnosed incidentally [4]. However, it can be misdiagnosed as pneumonia because of its presentation on chest X-ray as an opacity or lobar consolidation.

We are presenting a case of a 9-month-old boy with ectopic thoracic kidney associated with Bochdalek hernia which was misdiagnosed initially as an unresolved pneumonia. The aim of this case report is to shed light on this congenital anomaly and its rare presentation to help early diagnosis.

## Case presentation

**Figure 1:**
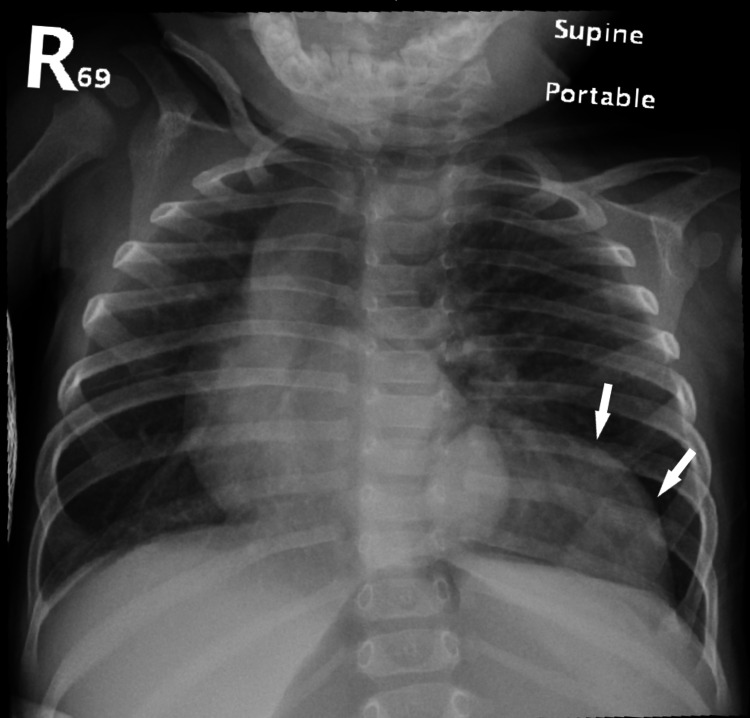
Chest radiography Chest X-ray showing left lower lung opacity (arrows).

A 9-month-old boy, who was diagnosed in the neonatal period with developmental dysplasia of the hip (DDH), hypospadias and bicuspid aortic valve, presented to the Emergency Department with three days history of cough, shortness of breath and fever associated with vomiting and diarrhea. History revealed that he was born at full term, and in view of family history of DDH and congenital heart disease in his siblings, he underwent hip ultrasound and cardiac echocardiography in the neonatal period with the above findings. He otherwise had an uneventful neonatal period with no NICU admission required. The mother reported a history of on and off respiratory symptoms for one month prior to this presentation. Upon examination, the patient was alert but mildly dehydrated and in respiratory distress with intercostal retractions with oxygen saturation of 94% on room air. Complete blood count was normal and renal function test showed normal creatinine (30 umol/l). Chest X-ray showed left lower lung opacity. He was treated as a case of bacterial pneumonia with intravenous ceftriaxone, and upon the clinical improvement of his presenting symptoms, he was discharged home to complete an oral antibiotics course with OPD follow-up. One week later, the patient presented with similar symptoms of fever, cough and increase work of breathing. Chest X-ray revealed persistent left lower lung opacity (Figure 1).

**Figure 2:**
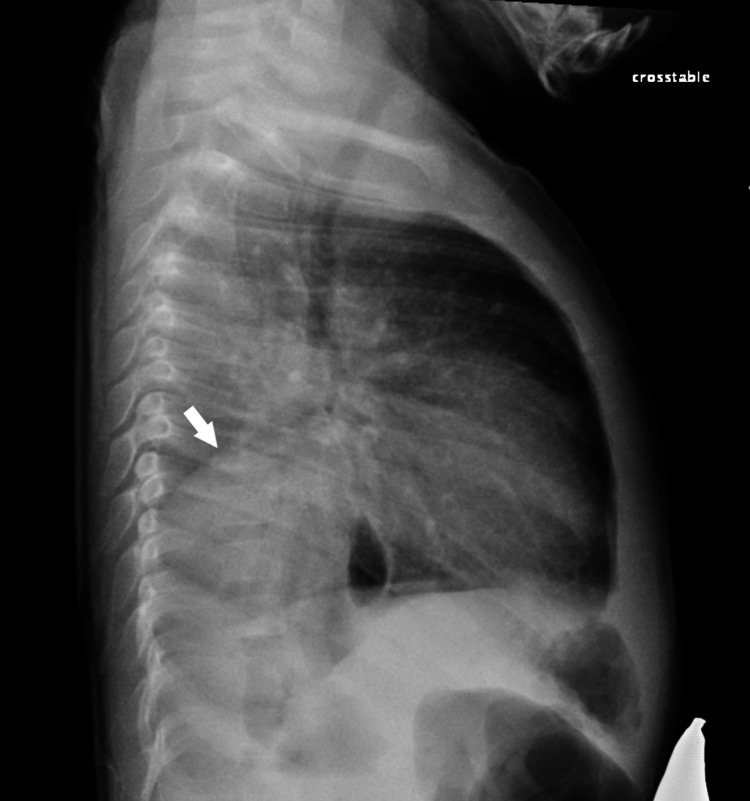
Lateral chest radiography Lateral chest X-ray showing opacity in the lower lobes posteriorly (arrow).

He was admitted as a case of unresolved pneumonia and was started on intravenous ceftriaxone. Three days later, there was no clinical improvement. Lateral chest X-ray showed opacity in the lower lobes posteriorly (Figure 2).

**Figure 3:**
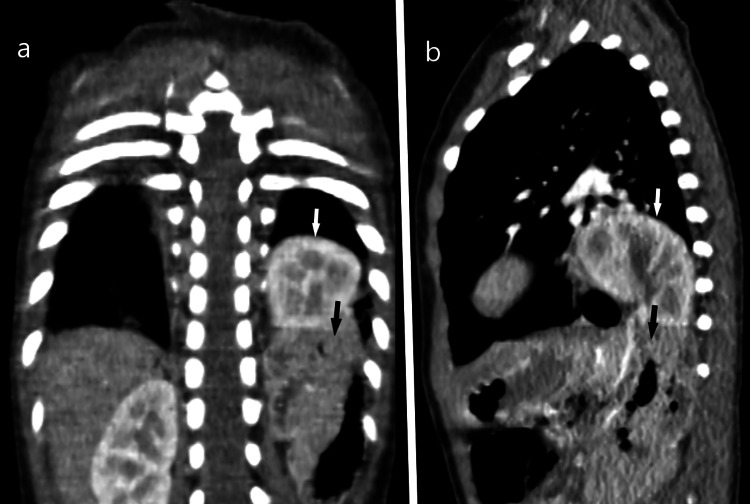
Coronal plane (a) and sagittal plane (b) showing herniation of the left kidney (white arrows) and parts of the large bowel loops (black arrows)

His images were reviewed by our pediatric radiologist, and he recommended to do a chest CT scan to rule out the possibility of congenital lung pathology. The chest CT scan revealed a non-visualization of the left posterior diaphragm with herniation of the left kidney and parts of the large bowel loops (Figure 3).

After an assessment by our pediatric surgical team, he was scheduled for an elective left diaphragmatic hernia repair four weeks later. The surgery was planned to be done laparoscopically but was converted to an open repair via a left subcostal incision due to the large size of the hernia sac. During the operation, the defect (Bochdalek hernia) was identified in the posterolateral edge of the diaphragm, on the left side, with associated herniation of the left kidney, pancreas and colon. All herniated structures were pushed down to proper intrabdominal location and nephropexy was performed. The surgery was uneventful and the patient was discharged five days later. A follow-up chest X-ray, two months later, in the pediatric surgery clinic showed normal diaphragmatic alignment. For the following four years, the child had no further chest infection-related admissions with normal renal function on follow-up laboratory test.

## Discussion

Ectopic kidneys are considered a rare anomaly in general, with a reported intrathoracic location in less than 5% of all kidney ectopias. Most cases of thoracic kidneys are asymptomatic and are diagnosed incidentally during adulthood. The prevalence is much lower in children, and about 39 pediatric intrathoracic ectopic kidney cases have been reported since 1985 [1,3].

A kidney is considered intrathoracic when it ascends above the level of the diaphragm into the posterior mediastinum. The pathophysiology of the intrathoracic kidney is still unclear. Normally, the kidney ascends to the level of L1 at the 8th week of gestation. Thoracic ectopia is believed to be due to early ascent of the kidney or late closure of the diaphragm [4]. Although a thoracic kidney may have a deformed shape, abnormal rotation, an elongated ureter, a high origin of renal vessels, and medial deviation of the lower pole of the kidney, it is usually fully functional, most cases maintain normal function and display no dysplastic parenchymal changes [3].

Intrathoracic ectopic kidneys have been classified into four sub-types with different associations and approach to management: (a) closed diaphragm, (b) eventration of the diaphragm, (c) diaphragmatic hernia, either congenital or acquired and (d) traumatic rupture of the diaphragm. The first two groups are usually asymptomatic, while the latter two are often symptomatic and require surgical intervention [5].

The incidence of the thoracic kidney associated with a congenital diaphragmatic hernia is reported to be <0.25% [6]. Our patient had an associated congenital diaphragmatic hernia and had recurrent respiratory symptoms which mandated an elective surgical resection.

The diagnosis of intrathoracic kidney could be challenging. Therefore, physicians should have a high index of suspension regarding any patient presenting with recurrent chest symptoms with the absence of one kidney at its normal position on ultrasonography.

Chest X-ray is the initial suggested modality for diagnosis. Ultrasonography, CT scan, magnetic resonance imaging (MRI), MR-urography and dimercaptosuccinic acid (DMSA) scintigraphy are other useful modalities that can be used to confirm the diagnosis of thoracic ectopic kidney. In our case, the persistent lung opacity raised our suspicion of a congenital lung pathology. Intrathoracic ectopic kidney was not on our initial list of differential diagnosis. The diagnosis was established following chest CT.

Regarding the management, most of the cases of intrathoracic kidney do not require treatment. Conservative therapy with regular follow-up is advocated for an asymptomatic, non-complicated, isolated anomaly [7-8]. For symptomatic children, who mostly present with recurrent respiratory symptoms and associated with diaphragmatic defect and herniation of the abdominal contents, surgical intervention will be needed [3]. In such a scenario, like our case, hernia repair and nephropexy are recommended [3].

## Conclusions

Congenital ectopic kidney is a rare clinical anomaly with the rarest presentation being in the thorax. It is a diagnostic challenge to physicians. To avoid unnecessary investigations and misdiagnosis, knowledge of this clinical entity is important. Intrathoracic ectopic kidney should be considered in the differential diagnosis of thoracic masses. Various imaging studies could be used to help diagnose and confirm a thoracic ectopic kidney. Treatment is usually conservative except when there are other associated anomalies like congenital diaphragmatic hernia where corrective surgery is required.
